# Presymptomatic geographical distribution of ALS patients suggests the involvement of environmental factors in the disease pathogenesis

**DOI:** 10.1007/s00415-023-11888-8

**Published:** 2023-07-25

**Authors:** Rosario Vasta, S. Callegaro, S. Sgambetterra, S. Cabras, F. Di Pede, F. De Mattei, E. Matteoni, M. Grassano, A. Bombaci, G. De Marco, G. Fuda, G. Marchese, F. Palumbo, A. Canosa, L. Mazzini, F. De Marchi, C. Moglia, U. Manera, A. Chiò, A. Calvo

**Affiliations:** 1 ALS Center, Department of Neuroscience “Rita Levi Montalcini”, University of Turin, Via Cherasco 15, 10126 Turin, Italy; 2 Department of Neuroscience “Rita Levi Montalcini”, University of Turin, Turin, Italy; 3 International School of Advanced Studies, University of Camerino, Camerino, Italy; 4 Department of Neuromuscular Diseases, Queen Square Institute of Neurology, UCL, London, WC1N 3BG UK; 5 Neurology 1, AOU Città della Salute e della Scienza di Torino, Turin, Italy; 6 Institute of Cognitive Science and Technologies, National Research Council, Rome, Italy; 7 ALS Center, Department of Neurology, Azienda Ospedaliero Universitaria Maggiore della Carità, and University of Piemonte Orientale, Novara, Italy

**Keywords:** Amyotrophic lateral sclerosis, Epidemiology, Etiology, Environment, Geoepidemiology, Spatial epidemiology

## Abstract

**Background:**

Given that the pathogenetic process of ALS begins many years prior to its clinical onset, examining patients’ residential histories may offer insights on the disease risk factors. Here, we analyzed the spatial distribution of a large ALS cohort in the 50 years preceding the disease onset.

**Methods:**

Data from the PARALS register were used. A spatial cluster analysis was performed at the time of disease onset and at 1-year intervals up to 50 years prior to that.

**Results:**

A total of 1124 patients were included. The analysis revealed a higher-incidence cluster in a large area (435,000 inhabitants) west of Turin. From 9 to 2 years before their onset, 105 cases were expected and 150 were observed, resulting in a relative risk of 1.49 (*P* = 0.04). We also found a surprising high number of patients pairs (51) and trios (3) who lived in the same dwelling while not being related. Noticeably, these occurrences were not observed in large dwellings as we would have expected. The probability of this occurring in smaller buildings only by chance was very low (*P* = 0.01 and *P* = 0.04 for pairs and trios, respectively).

**Conclusions:**

We identified a higher-incidence ALS cluster in the years preceding the disease onset. The cluster area being densely populated, many exposures could have contributed to the high incidence ALS cluster, while we could not find a shared exposure among the dwellings where multiple patients had lived. However, these findings support that exogenous factors are likely involved in the ALS pathogenesis.

**Supplementary Information:**

The online version contains supplementary material available at 10.1007/s00415-023-11888-8.

## Introduction

The causes of amyotrophic lateral sclerosis (ALS) are largely unknown although approximately 20% of cases have been attributed to mutations in one of about 30 genes. For the remaining cases, it is believed that a combination of genetic and environmental factors may contribute to the development of the disease [1].

Obtaining reproducible evidence on ALS environmental risk factors has proven to be challenging for several reasons, including data availability and variations in the use of proxies. Cigarette smoking, physical activity, and dyslipidemia have been recognized as de facto risk factors for the development of ALS. However, studies on heavy metals, pesticides, electromagnetic fields, certain occupations, drugs, and cyanotoxins have yielded inconsistent results, and further investigation is needed to establish their role in the disease pathogenesis [1].

Analyzing the geographical distribution of patients could reveal areas in which the disease incidence is higher or lower than expected, eventually providing hints on environmental or genetic risk factors. As a result, numerous studies worldwide have assessed the geoepidemiology of ALS, often revealing high-incidence clusters and suggesting potential environmental risk factors [2, 3]. We also contributed by examining the distribution of ALS patients in Piemonte and Valle d’Aosta, Italy, and demonstrating its substantial homogeneity [4, 5]. Most of these reports focused solely on the location of patients at the time of their diagnosis, while it is now well-established that the pathogenesis of ALS begins years before its clinical onset [6, 7]. As patients may have lived elsewhere during the years preceding their diagnosis, limiting the analysis to a single time point could be misguiding [8].

Here, we analyzed the geographical distribution of a large population-based cohort of ALS patients during the 50 years preceding their disease onset.

## Methods

All patients included in the Piemonte and Valle d’Aosta ALS Register (PARALS), who were diagnosed with ALS from 2007 to 2014, were considered. The PARALS inclusion criteria have been previously described in detail [9]. The study area for the register includes the regions of Valle d’Aosta and Piemonte but only patients who were residents in Piemonte at the time of their diagnosis were considered for this study. Piemonte is located in the Northwest of Italy and covers an area of about 25,400 km^2^. According to the 2011 census, the region comprises 1180 municipalities with a total population of 4,416,475 residents, of which 2,133,075 were males [10].

Using registries from Piemonte municipalities, the residential history of each patients was collected from time of disease onset and up to 50 years prior to that. Then, we analyzed their geographical locations going back in time at 1-year intervals from the disease onset. As the disease onsets were asynchronous, we calculated the populations at risk for each of these time points by taking the average of populations from the different years considered. At each time point, a cluster analysis was then performed using the Kulldorff’s statistics.

Kulldorff’s analysis proceeds by superimposing circles of variable radius—from zero to the one including 25% of the overall population—centered on each municipality centroid. The incidence inside and outside each circle is compared. To assess the statistical significance, a Monte Carlo simulation is then conducted, in which patients are randomly distributed throughout the study area for 999 iterations. The likelihood ratio test is used as the statistical test, and the p value is set at 0.05 [11].

Analyses using the resident populations from 1982 to 2014 were sex- and age-adjusted (using the age-classes: 0–24, 25–34, 35–44, 45–54, 55–64, 65–74, 75–84 and ≥ 85). Analyses prior to 1981 (i.e., from 15 years before onset) were not sex- and age-adjusted because of the unavailability of reference data. Additionally, prior to 1981, the overall population count was available only for the census years (1951, 1961, 1971 and 1981) [10]. For the years in between, population counts were imputed by evenly distributing the difference within each interval.

While collecting data, we noticed that some dwellings appeared twice or three times. Although adjusting the administrative scale could affect the results of cluster analysis (so-called Modifiable Unit Area Problem) [12], these occurrences would have been too granular to be captured. Thus, to address this issue, we performed a Monte Carlo simulation to assess their statistical significance.

In 2011, Torino had 39,184 dwellings occupied by 870,474 residents. A total of 373 patients wild-type for major ALS genes had lived in Turin at least once during their residential history and changed their address an average of 3.6 times. To simulate this, each Monte Carlo iteration randomly distributed 373 patients into 39,184 virtual positions over 3.6 consecutive rounds, considering the resident capacity of each dwelling and ensuring that no patient occupied the same dwelling twice in the same iteration. A total of 2000 iterations were launched and for each the number of patients who shared a dwelling in pairs or in triples were calculated. Finally, the probability distributions of double and triple occurrences were created.

Kulldorff analysis was performed using the SatScan analysis v9.4.4. R 3.4 software (4.0.5 version) [13] was used to perform basic statistics, figures, and data preparation for SatScan analysis [14].

Phenotypes at diagnosis were defined according to a previous classification [15].

When information was available, genetics analysis consisted in the amplification of all the coding exons and 50 bp of the flanking intron–exon boundaries of *SOD1*, of exon 6 of *TARDBP* and of exons 14 and 15 of *FUS* via polymerase chain reaction (PCR). The amplified sequences were then sequenced using the Big-Dye Terminator v3.1 sequencing kit (Applied Biosystems Inc.) and run on an ABIPrism 3130 genetic analyzer. These exons were selected as the vast majority of known pathogenic variants are located within these mutational hotspots. A repeat-primed PCR assay was used to detect the presence of the GGGGCC hexanucleotide expansion in the first intron of *C9ORF72*.

The study was approved by the Ethical Committee of the Turin ALS Center (Comitato Etico Azienda Ospedaliero-Universitaria Città della Salute e della Scienza, Torino).

Patients provided written informed consent before enrollment. The databases were anonymized according to the Italian law for the protection of privacy.

## Results

We included in the study 1124 patients whose demographical and clinical characteristics were consistent with those reported in previous population-based studies (Table 1). Their 50-year residential history was reconstructed with missing data ranging from 0.62% at disease onset to 10.5% forty years before and 19.7% fifty years prior to disease onset (supplementary Fig. 1).

**Table 1:** Demographical and clinical characteristics of the population included in the study, overall and stratified by residence inside and outside the higher-incidence cluster

	Overall population (*n* = 1124)	Patients outside the cluster (*n* = 974)	Patients inside the cluster (*n* = 150)	*p* value
Sex, *M* (%)	598 (53.2)	511 (52.5)	87 (58.0)	0.24
Onset site (%)				0.38
Bulbar	395 (35.1)	349 (35.8)	46 (30.7)	
Respiratory	21 (1.9)	19 (2.0)	2 (1.3)	
Spinal	708 (63.0)	606 (62.2)	102 (68.0)	
Onset age (median [IQR])	68.19 [60.22–74.38]	68.44 [60.21–74.56]	66.77 [61.05–73.41]	0.23
Diagnostic delay (median [IQR])	9.10 [5.10–13.20]	9.10 [5.10–13.20]	9.10 [6.03–12.93]	0.80
Genetics, positive (%)				
C9ORF72	64 (5.7)	54 (5.5)	10 (6.7)	0.35
FUS	6 (0.5)	4 (0.4)	2 (1.3)	
SOD1	19 (1.7)	17 (1.7)	2 (1.3)	
TARDBP	14 (1.2)	12 (1.2)	2 (1.3)	
Wild type	810 (72.1)	696 (71.5)	114 (76.1)	
Missing data	211 (18.8)	191 (19.7)	20 (13.3)	
Phenotype at diagnosis (%)				
Bulbar	395 (35.1)	349 (35.8)	46 (30.7)	0.80
Classic	335 (29.8)	286 (29.4)	49 (32.7)	
Flail arm	65 (5.8)	56 (5.7)	9 (6.0)	
Flail leg	207 (18.4)	179 (18.4)	28 (18.6)	
Prevalent upper motor neuron	101 (9.0)	85 (8.7)	16 (10.7)	
Respiratory	21 (1.9)	19 (2.0)	2 (1.3)	

In-cluster patients were selected considering the 9 years prior to disease onset time point, i.e. the earliest in which the cluster appeared as significant

Phenotypes at diagnosis were defined according to a previous classification

*IQR* interquartile range

**Fig. 1:**
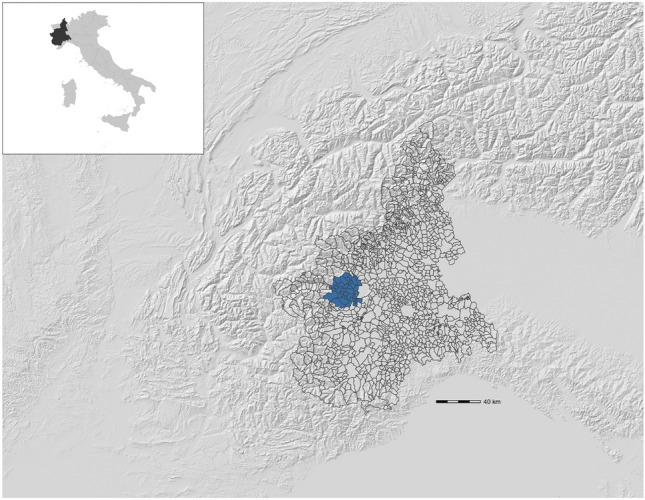
The higher-incidence ALS cluster (blue) identified in the western region of Piemonte, between 2 to 9 years before patients’ clinical onset. The original map has been provided by Arpa Piemonte (Regional Environmental Protection Agency, Agenzia Regionale per la Protezione Ambientale) and adapted to the purpose of the work

Most patients (1117, 99.4%) did not change their address between disease onset and diagnosis. In line with our previous report using data at diagnosis [4], the analysis conducted at the disease onset did not identify any clusters in the study area. However, when analyzing years prior to disease onset, we were able to identify a cluster of higher-incidence in a northwestern region of Piemonte (Fig. 1). In the years from 2 to 9 prior to their disease onset, 50% more patients than expected lived in an area of approximately 435,000 inhabitants. Nine years before their onset, 105 presymptomatic cases were expected in this area but 150 were observed, resulting in a relative risk (RR) of 1.49 (*P* = 0.04). The majority of these patients (143) remained within the area throughout the entire 9–2 years before the onset period, along with an additional 7 patients whose presence varied during the mentioned period. One year before the onset of patients, while the number of observed cases remained unchanged, the cluster population (and the expected number of cases) slightly increased, enough to make the cluster no more significant. Supplementary Table 1 provides the results from this cluster, as well as data from non-significant clusters detected by the analysis. Patients in the cluster had similar demographical and clinical characteristics to the overall population (Table 1). No lower-incidence clusters were detected.

In our database, 78 addresses recurred twice and four addresses recurred three times. Since most of these occurrences (70, 89.7%) were located in Turin, we performed a restricted analysis focusing on this city. Our interest was in identifying shared environmental risk factor among patients who had lived in the same dwelling. Therefore, all occurrences in which at least one patient had a known genetic cause were excluded. Specifically, we excluded 3 occurrences in which patients were relatives (and genetic cases) and 13 more addresses where one patient in each was positive for a mutation/expansion in one of the four major genes. This totaled 54 addresses where more than one patient had lived while not being relatives. Out of these, 51 dwellings had housed two different patients each, while 3 buildings had housed three different patients each. In 25 out of the 51 dwellings, patients had resided at the same time for a median duration of 51.5 months (interquartile range, IQR 27.3–90.0).

According to the Monte Carlo simulations, the likelihood of two patients living in the same dwelling and this occurring in 51 distinct dwellings was 0.70, while the probability of 3 triple occurrences was 0.86. Surprisingly, however, most of these addresses did not correspond to large dwellings as we would have expected with a random distribution. Accordingly, we repeated the Monte Carlo iterations by reallocating the patients only in dwellings of similar size to those with multiple occurrences. By restricting the simulation to dwellings smaller than the largest one where two or three patients had lived (resident capacity of 254), the probability of observing 51 buildings where two patients had lived in each only by chance significantly decreased (*P* = 0.49). Similarly, the probability of finding three buildings, each inhabited by three different patients, was also lower (*P* = 0.40). The analysis was then further carried out by stratifying based on the size of dwellings with multiple occurrences, which was determined as their residents’ capacity. For example, the 20% largest buildings in which double occurrences were observed corresponded in terms of their size to only 5.6% of the total number of buildings. By removing these large buildings, we would have found 41 dwellings where two patients had resided, and the probability of this event happening by chance was very low (*P* = 0.01). Similarly, by excluding the highest quartile in the simulation for triple occurrences, the probability of finding three dwellings, each inhabited by three different patients, was also unlikely (*P* = 0.04). The probabilities further decreased when larger buildings were excluded (supplementary table 2 and table 3).

## Discussion

By examining the residential histories of about 1100 ALS patients diagnosed in Piemonte, Northern Italy, we found that a larger than expected number of patients (50% more) had lived in an area west of Turin in the 2–9 years preceding their clinical onset (105 expected versus 150 observed). The percentage of mutations in ALS causal genes among these patients was comparable to that of the out-of-cluster cases, which suggests that some environmental factors could have increased the ALS risk among the area inhabitants. Such exposure(s) could have been only detrimental to individuals who were already susceptible to the disease due to genetics and/or other environmental exposures. Moreover, since most in-cluster patients had lived in the area for the entire period during which the excess of cases was significant, we can speculate that a prolonged exposure was required to increase the risk of ALS.

The cluster occurred in one of the most highly populated area in Piemonte, making it difficult to point at a single perpetrator. In the city of Turin as in the surroundings municipalities within the cluster area, the air pollution reaches its highest levels [16]. Airborne particles get to the olfactory bulb and may be small enough to pass the blood brain barrier, reaching more distal areas of the central nervous system such as the frontal cortex. Here, these particles could initiate a chronic inflammation leading to an oxidative stress that over time will damage various cellular components [17]. Accordingly, it has been previously suggested that individuals more exposed to air pollutants could have a higher ALS risk [17–19].

The power line network in the cluster area is also particularly dense, resulting in higher levels of extremely low-frequency electromagnetic fields [16]. The extent to which electromagnetic fields could increase the risk of developing ALS remains uncertain. Some studies conducted on cellular and animal models of ALS have suggested that electromagnetic fields may impair antioxidant defense mechanisms [20]. However, while some epidemiological studies confirmed this relationship, others did not [21], and a recent meta-analysis of six studies concluded that there is scant evidence linking the exposure to electromagnetic fields with an increased risk of ALS [19]. Our recent study also demonstrated that this exposure does not alter neither the disease progression nor the onset age [22]. Nevertheless, other exposures typical of urban and densely populated, such as diet, physical exercise, behavioral or overall lifestyle [23], could also contribute to the elevated risk of ALS in that area [17], thus acting as a potential confounder. Therefore, while environment is more likely to underlie this finding, it is more difficult to identify a specific environmental factor.

We also found a surprising high number of patients pairs (51) and trios (3) who were not related but still had lived in the same building or house in Turin. Given the large number of dwellings and the number of patients who lived in Turin at some point in their lives, it was likely to encounter these occurrences. However, it was highly unlikely to observe them in smaller dwellings at it was in our case. While it is possible that random genetics co-occurrence could explain these observations, it is more likely that they were the results of a shared environmental exposure. It is possible to speculate that metals released by water pipes could have increase the ALS risk among the inhabitants of these buildings [24], particularly if maintenance had not been carried out regularly. However, despite consulting historical archives, this hypothesis could not be confirmed, neither other potential factors could be identified. This task is further complicated considering that at least some of these occurrences may have been due to chance alone. Nonetheless, even if we cannot pinpoint a specific exposure, the fact that such an improbable event occurred further suggests that environmental factors likely play a role in ALS pathogenesis.

Geoepidemiological studies have been conducted in the field of ALS research for some time now [2]. Higher-incidence clusters of ALS have been described in many countries and linked to the exposure to L-BMAA [25–28], metals [29–32], mycotoxins [33] or pesticides [34, 35]. Some other studies have reported a uniform distribution of ALS cases [4, 5] or even low-incidence areas, attributed to a different genetic admixture [36].

Some anecdotal reports have also described unexpected geographic occurrences of ALS [37]. For instance, conjugal cases have been repeatedly described [37–39], despite spouses are likely to share many exposures beyond their residency. In 1974, it was reported that three unrelated patients living within one block developed the disease within a short time from each other [40]. A few years later, the case of three ALS patients who lived within one-block radius in a Boston suburban was reported [41]. Another report documented four unrelated ALS cases occurring in a 10-year period in a small county with 4000 inhabitants in South Dakota. Three of these cases lived less than 3 km apart their entire lives [32]. In Montreal, three unrelated individuals of Ashkenazi Jewish extraction living in the same building developed ALS within an 18-month period [41].

While these reports are mostly based on patients’ location at the time of their diagnosis, we know now that ALS pathogenetic process begins many years before its clinical onset. During this preclinical period, multiple adaptive processes likely allow for the functionality of neuronal pathways despite their ongoing damage, until a critical threshold is reached [6, 7, 42]. Similar to certain cancers, the exposure to causal factors and the clinical onset could be delayed by a period of decades [43]. Therefore, studies that rely solely on patients’ distribution at the time of diagnosis may miss crucial information about the genetic and environmental risk factors.

Our study is among the few that have utilized residential history data. Through the analysis of the residential histories of ALS patients in the USA, it was found that long-term exposure (5 years) to airborne toxics, such as styrene, chromium, nickel and acid nitric, could increase the risk of developing ALS, with odds ratio ranging from 1.12 to 1.36 [44]. Another study examined residential records of 500 ALS patients and nearly 2000 controls in Ohio, for the 5 years leading up to a survey. The results revealed that individuals who resided in counties with increased usage of herbicides, glyphosate and certain insecticides had an higher risk of developing ALS (OR from 1.25 to 1.32) [45]. Similarly, in Michigan, researchers administered a detail questionnaire to both cases and controls, also obtaining their residential and occupational histories. The results showed ALS patients had a higher likelihood of being exposed to fertilizers to treat private yards and gardens and also had higher occupational exposure to pesticides compared to the control group. This latter finding was further confirmed in a study involving a larger cohort [46, 47]. In a case–control study conducted in Italy, authors retrieved the residential histories from 35 years prior to diagnosis and found that consumption of drinking water with higher levels of inorganic selenium could increase the risk of developing ALS (relative risk 5.4, 95% CI 1.1–26) [48]. Another interesting Finnish study used historical information to compare cases and controls who lived in the same birthplace area but moved differently thereafter. By comparing the mobility of movers and stayers, the authors found that cases were more likely to remain in their birthplace, suggesting the potential pathogenetic role of environmental exposures despite being well aware of numerous possible confounders [8].

While retrieving residential histories can be time-consuming, it can be worth the effort as it can offer valuable insights. By analyzing residential histories, we can obtain a more precise estimate of an individual’s lifetime exposure to certain toxics, identify space–time clusters or, as it is the case of our study, reveal clusters that may have otherwise been missed [49]. Additionally, we were able to systematically examine the unexpected occurrence of unrelated patients who had lived in the same building, rather than relying on anecdotal reports from patients or observations by physicians.

However, it is important to note that the residential history alone does not provide a complete picture of an individual’s exposure and that professional exposures should be also considered.

Furthermore, we only included patients who were resident in Piemonte at the time of their diagnosis, while some patients may have lived in Piemonte before the diagnosis but were residing elsewhere when diagnosed. This could have impacted the cluster analysis in either direction, while could have led to an underestimation of the number of patients living in the same dwelling. Also, while being widely used to detect spatial clusters, Kulldorff scan statistics is not without limits (for example, the search for irregularly shaped clusters) and other statistics have been also developed [50–52]. Lastly, it is important to note that the genetics cannot be disregarded in the ALS etiology [1]. Although it would be challenging, grouping patients according to genetic predispositions could provide a more powerful means to investigate the differential impact of various environmental exposures in the disease pathogenesis.

While being aware of these limitations, this analysis has also several strengths. Firstly, we used a large cohort of patients, which allowed for more robust and reliable results. Additionally, patients were deeply phenotyped adding valuable information on cases included in the geographical occurrences we identified. Finally, we were able to reconstruct most of patients’ lifetime residential history.

In conclusion, the study revealed a higher-incidence cluster of ALS patients 2 to 9 years prior to their onset and that subsequently disappeared. Moreover, we found an unexpected high number of patients who had lived in pairs or triples in the same dwelling. Although we were not able to identify a specific factor associated to these findings, it is likely that these results were justified by some exogenous exposures. This provides further evidence that environmental factors are involved and likely play a necessary role in the pathogenesis of most ALS cases. Future studies considering genetics and environmental factors will potentially provide critical insights into the puzzle of ALS causes.

### Supplementary Information

Below is the link to the electronic supplementary material.

Supplementary file1 Supplementary figure 1. Residential histories completeness over the 50 years prior to patients’ disease onset, displayed as percentage of the total study population (JPG 2707 KB)

Supplementary file2 (DOCX 79 KB)

Supplementary file3 (DOC 40 KB)

Supplementary file4 (DOC 40 KB)

## Data Availability

Due to the use of sensitive data, specifically residence addresses from each patient, we chose not to disclose the data.
