# Supplementary figures and images for: Presymptomatic geographical distribution of ALS patients suggests the involvement of environmental factors in the disease pathogenesis
Source: J Neurol. 2023 Jul 25;270(11):5475–82. doi: 10.1007/s00415-023-11888-8 (PMC10576667; doi:10.1007/s00415-023-11888-8)

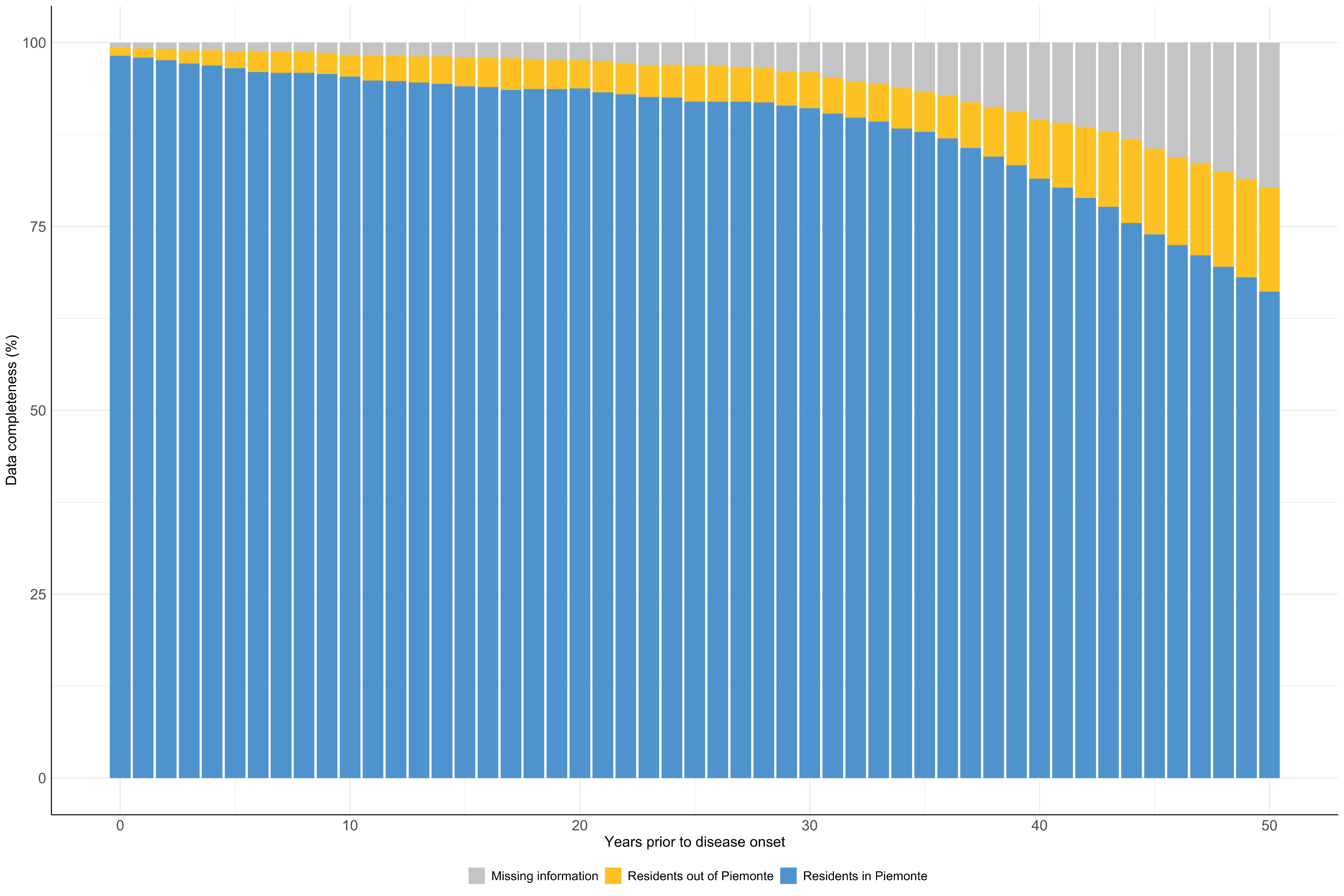

Supplement: Supplementary file 1 — Supplementary file1 Supplementary figure 1. Residential histories completeness over the 50 years prior to patients’ disease onset, displayed as percentage of the total study population (JPG 2707 KB) [file 415_2023_11888_MOESM1_ESM.jpg]
