# Supplementary material for: Presymptomatic geographical distribution of ALS patients suggests the involvement of environmental factors in the disease pathogenesis
Source: J Neurol. 2023 Jul 25;270(11):5475–82. doi: 10.1007/s00415-023-11888-8 (PMC10576667; doi:10.1007/s00415-023-11888-8)
Supplement: Supplementary file 2 — Supplementary file2 (DOCX 79 KB) [file 415_2023_11888_MOESM2_ESM.docx]

**Supplementary Table 1.** Characteristics of clusters identified using the SatScan Analysis and arranged by years before patients’ onset. Bold refers to the significant high-incidence clusters revealed by the analysis.

| **Years before onset** | **Communalities included** | | **Resident population** | | **Expected cases** | | **Observed cases** | | **Relative Risk** | | **P-value** | |
| --- | --- | --- | --- | --- | --- | --- | --- | --- | --- | --- | --- | --- |
| 0 | 39 | 451731 | | 108.8 | | 152 | | 1.46 | | 0.11 | |  |
| 0 | 117 | 250922 | | 67.2 | | 38 | | 0.55 | | 0.2 | |  |
| 0 | 26 | 27890 | | 7 | | 0 | | 0 | | 0.38 | |  |
| 0 | 22 | 21356 | | 5.5 | | 0 | | 0 | | 0.84 | |  |
| 0 | 2 | 2794 | | 0.8 | | 5 | | 6.15 | | 0.95 | |  |
| 0 | 8 | 21089 | | 5 | | 0 | | 0 | | 0.96 | |  |
| 0 | 76 | 123363 | | 31.5 | | 16 | | 0.5 | | 0.98 | |  |
| 0 | 3 | 896 | | 0.3 | | 3 | | 11.33 | | 0.98 | |  |
| 0 | 7 | 33443 | | 7.7 | | 17 | | 2.24 | | 0.99 | |  |
| 0 | 2 | 6470 | | 1.4 | | 6 | | 4.37 | | 1 | |  |
| 1 | 39 | 449474 | | 108.4 | | 153 | | 1.48 | | 0.07 | |  |
| 1 | 117 | 250512 | | 67.1 | | 38 | | 0.55 | | 0.23 | |  |
| 1 | 26 | 27826 | | 6.9 | | 0 | | 0 | | 0.43 | |  |
| 1 | 22 | 21423 | | 5.5 | | 0 | | 0 | | 0.87 | |  |
| 1 | 2 | 2811 | | 0.8 | | 5 | | 6.17 | | 0.93 | |  |
| 1 | 8 | 21001 | | 5 | | 0 | | 0 | | 0.95 | |  |
| 1 | 3 | 899 | | 0.3 | | 3 | | 11.39 | | 0.98 | |  |
| 1 | 76 | 122946 | | 31.5 | | 16 | | 0.5 | | 0.98 | |  |
| 1 | 7 | 33153 | | 7.6 | | 17 | | 2.25 | | 0.99 | |  |
| 1 | 2 | 6378 | | 1.4 | | 6 | | 4.41 | | 0.99 | |  |
| 1 | 27 | 13299 | | 4 | | 0 | | 0 | | 1 | |  |
| **2** | **39** | **447218** | | **108** | | **153** | | **1.48** | | **0.05** | |  |
| 2 | 94 | 232808 | | 61.9 | | 35 | | 0.55 | | 0.36 | |  |
| 2 | 26 | 27761 | | 6.9 | | 0 | | 0 | | 0.47 | |  |
| 2 | 22 | 21494 | | 5.5 | | 0 | | 0 | | 0.89 | |  |
| 2 | 2 | 2825 | | 0.8 | | 5 | | 6.2 | | 0.94 | |  |
| 2 | 8 | 20897 | | 4.9 | | 0 | | 0 | | 0.96 | |  |
| 2 | 3 | 902 | | 0.3 | | 3 | | 11.44 | | 0.98 | |  |
| 2 | 76 | 122523 | | 31.4 | | 16 | | 0.5 | | 0.98 | |  |
| 2 | 7 | 32869 | | 7.6 | | 17 | | 2.26 | | 0.99 | |  |
| 2 | 2 | 6274 | | 1.4 | | 6 | | 4.45 | | 0.99 | |  |
| **3** | **39** | **444927** | | **107.4** | | **153** | | **1.49** | | **0.04** | |  |
| 3 | 94 | 232591 | | 61.6 | | 35 | | 0.55 | | 0.35 | |  |
| 3 | 26 | 27718 | | 6.9 | | 0 | | 0 | | 0.4 | |  |
| 3 | 22 | 21535 | | 5.5 | | 0 | | 0 | | 0.87 | |  |
| 3 | 2 | 2841 | | 0.8 | | 5 | | 6.22 | | 0.95 | |  |
| 3 | 8 | 20781 | | 4.9 | | 0 | | 0 | | 0.97 | |  |
| 3 | 3 | 902 | | 0.3 | | 3 | | 11.52 | | 0.98 | |  |
| 3 | 76 | 122057 | | 31.2 | | 16 | | 0.51 | | 0.98 | |  |
| 3 | 7 | 32599 | | 7.5 | | 17 | | 2.28 | | 0.99 | |  |
| 3 | 2 | 6169 | | 1.3 | | 6 | | 4.5 | | 0.99 | |  |
| 3 | 16 | 26291 | | 6.9 | | 1 | | 0.14 | | 0.99 | |  |
| 3 | 27 | 13288 | | 3.9 | | 0 | | 0 | | 0.99 | |  |
| **4** | **39** | **442536** | | **107** | | **155** | | **1.52** | | **0.02** | |  |
| 4 | 94 | 232386 | | 61.4 | | 35 | | 0.56 | | 0.44 | |  |
| 4 | 16 | 26289 | | 6.9 | | 0 | | 0 | | 0.47 | |  |
| 4 | 26 | 27671 | | 6.8 | | 0 | | 0 | | 0.48 | |  |
| 4 | 22 | 21572 | | 5.5 | | 0 | | 0 | | 0.84 | |  |
| 4 | 7 | 32337 | | 7.5 | | 18 | | 2.43 | | 0.85 | |  |
| 4 | 36 | 17680 | | 5.3 | | 0 | | 0 | | 0.87 | |  |
| 4 | 6 | 3768 | | 1.1 | | 6 | | 5.38 | | 0.88 | |  |
| 4 | 3 | 902 | | 0.3 | | 3 | | 11.67 | | 0.97 | |  |
| 4 | 8 | 20664 | | 4.9 | | 0 | | 0 | | 0.98 | |  |
| 4 | 2 | 6062 | | 1.3 | | 6 | | 4.55 | | 0.98 | |  |
| **5** | **39** | **439979** | | **106.6** | | **153** | | **1.51** | | **0.02** | |  |
| 5 | 94 | 232250 | | 61.1 | | 34 | | 0.54 | | 0.28 | |  |
| 5 | 26 | 27640 | | 6.8 | | 0 | | 0 | | 0.46 | |  |
| 5 | 13 | 21051 | | 5.6 | | 0 | | 0 | | 0.81 | |  |
| 5 | 22 | 21602 | | 5.5 | | 0 | | 0 | | 0.84 | |  |
| 5 | 6 | 3794 | | 1.1 | | 6 | | 5.42 | | 0.87 | |  |
| 5 | 36 | 17702 | | 5.3 | | 0 | | 0 | | 0.89 | |  |
| 5 | 3 | 905 | | 0.3 | | 3 | | 11.79 | | 0.97 | |  |
| 5 | 8 | 20550 | | 4.9 | | 0 | | 0 | | 0.97 | |  |
| 5 | 7 | 32083 | | 7.5 | | 17 | | 2.3 | | 0.98 | |  |
| 5 | 2 | 5959 | | 1.3 | | 6 | | 4.6 | | 0.99 | |  |
| 5 | 8 | 17194 | | 4.5 | | 0 | | 0 | | 0.99 | |  |
| **6** | **39** | **437456** | | **105.9** | | **153** | | **1.52** | | **0.02** | |  |
| 6 | 94 | 232184 | | 60.7 | | 34 | | 0.55 | | 0.28 | |  |
| 6 | 26 | 27637 | | 6.8 | | 0 | | 0 | | 0.42 | |  |
| 6 | 13 | 21063 | | 5.6 | | 0 | | 0 | | 0.85 | |  |
| 6 | 22 | 21622 | | 5.4 | | 0 | | 0 | | 0.86 | |  |
| 6 | 36 | 17726 | | 5.2 | | 0 | | 0 | | 0.92 | |  |
| 6 | 3 | 901 | | 0.2 | | 3 | | 12.01 | | 0.97 | |  |
| 6 | 8 | 20430 | | 4.8 | | 0 | | 0 | | 0.97 | |  |
| 6 | 7 | 31856 | | 7.4 | | 17 | | 2.31 | | 0.98 | |  |
| 6 | 2 | 5858 | | 1.3 | | 6 | | 4.66 | | 0.98 | |  |
| 6 | 8 | 17194 | | 4.5 | | 0 | | 0 | | 0.99 | |  |
| 6 | 1 | 326 | | 0.1 | | 2 | | 19.9 | | 1 | |  |
| **7** | **39** | **435210** | | **105.5** | | **150** | | **1.49** | | **0.04** | |  |
| 7 | 94 | 232247 | | 60.6 | | 33 | | 0.53 | | 0.19 | |  |
| 7 | 26 | 27613 | | 6.8 | | 0 | | 0 | | 0.42 | |  |
| 7 | 13 | 21077 | | 5.6 | | 0 | | 0 | | 0.8 | |  |
| 7 | 22 | 21633 | | 5.4 | | 0 | | 0 | | 0.84 | |  |
| 7 | 36 | 17749 | | 5.2 | | 0 | | 0 | | 0.91 | |  |
| 7 | 3 | 895 | | 0.2 | | 3 | | 12.16 | | 0.96 | |  |
| 7 | 8 | 20319 | | 4.8 | | 0 | | 0 | | 0.97 | |  |
| 7 | 7 | 31642 | | 7.4 | | 17 | | 2.32 | | 0.98 | |  |
| 7 | 2 | 5763 | | 1.3 | | 6 | | 4.7 | | 0.98 | |  |
| 7 | 8 | 17195 | | 4.5 | | 0 | | 0 | | 0.99 | |  |
| 7 | 1 | 328 | | 0.1 | | 2 | | 19.87 | | 0.99 | |  |
| **8** | **39** | **433360** | | **105.5** | | **149** | | **1.48** | | **0.07** | |  |
| 8 | 94 | 232551 | | 60.5 | | 33 | | 0.53 | | 0.21 | |  |
| 8 | 26 | 27604 | | 6.8 | | 0 | | 0 | | 0.43 | |  |
| 8 | 13 | 21089 | | 5.6 | | 0 | | 0 | | 0.8 | |  |
| 8 | 22 | 21656 | | 5.4 | | 0 | | 0 | | 0.84 | |  |
| 8 | 16 | 21387 | | 5.1 | | 14 | | 2.77 | | 0.87 | |  |
| 8 | 36 | 17783 | | 5.1 | | 0 | | 0 | | 0.94 | |  |
| 8 | 3 | 891 | | 0.2 | | 3 | | 12.37 | | 0.95 | |  |
| 8 | 8 | 20221 | | 4.8 | | 0 | | 0 | | 0.97 | |  |
| 8 | 3 | 8748 | | 2.3 | | 8 | | 3.56 | | 0.99 | |  |
| 8 | 1 | 330 | | 0.1 | | 2 | | 20.09 | | 0.99 | |  |
| 8 | 8 | 17191 | | 4.5 | | 0 | | 0 | | 1 | |  |
| 8 | 7 | 31471 | | 7.4 | | 16 | | 2.19 | | 1 | |  |
| **9** | **39** | **431606** | | **105.2** | | **150** | | **1.49** | | **0.04** | |  |
| 9 | 94 | 233052 | | 60.3 | | 33 | | 0.53 | | 0.23 | |  |
| 9 | 26 | 27504 | | 6.7 | | 0 | | 0 | | 0.46 | |  |
| 9 | 13 | 21062 | | 5.5 | | 0 | | 0 | | 0.84 | |  |
| 9 | 22 | 21600 | | 5.4 | | 0 | | 0 | | 0.87 | |  |
| 9 | 3 | 873 | | 0.2 | | 3 | | 12.78 | | 0.94 | |  |
| 9 | 36 | 17652 | | 5 | | 0 | | 0 | | 0.94 | |  |
| 9 | 8 | 20130 | | 4.8 | | 0 | | 0 | | 0.97 | |  |
| 9 | 16 | 21138 | | 5 | | 13 | | 2.6 | | 0.98 | |  |
| 9 | 3 | 8792 | | 2.3 | | 8 | | 3.56 | | 0.98 | |  |
| 9 | 8 | 17186 | | 4.5 | | 0 | | 0 | | 0.99 | |  |
| 9 | 1 | 326 | | 0.1 | | 2 | | 20.61 | | 0.99 | |  |
| 9 | 7 | 31285 | | 7.3 | | 16 | | 2.19 | | 1 | |  |
| 10 | 39 | 429250 | | 104.8 | | 148 | | 1.48 | | 0.09 | |  |
| 10 | 94 | 233555 | | 60 | | 32 | | 0.52 | | 0.21 | |  |
| 10 | 26 | 27410 | | 6.7 | | 0 | | 0 | | 0.52 | |  |
| 10 | 13 | 21024 | | 5.5 | | 0 | | 0 | | 0.87 | |  |
| 10 | 22 | 21543 | | 5.4 | | 0 | | 0 | | 0.89 | |  |
| 10 | 37 | 19558 | | 5.4 | | 0 | | 0 | | 0.89 | |  |
| 10 | 3 | 853 | | 0.2 | | 3 | | 13.3 | | 0.94 | |  |
| 10 | 8 | 20049 | | 4.8 | | 0 | | 0 | | 0.96 | |  |
| 10 | 16 | 20884 | | 5 | | 13 | | 2.64 | | 0.98 | |  |
| 10 | 8 | 17203 | | 4.4 | | 0 | | 0 | | 0.98 | |  |
| 10 | 3 | 8841 | | 2.2 | | 8 | | 3.57 | | 0.98 | |  |
| 10 | 1 | 322 | | 0.1 | | 2 | | 21.2 | | 1 | |  |
| 11 | 94 | 234049 | | 59.5 | | 31 | | 0.51 | | 0.1 | |  |
| 11 | 39 | 427000 | | 103.9 | | 146 | | 1.47 | | 0.13 | |  |
| 11 | 26 | 27430 | | 6.6 | | 0 | | 0 | | 0.51 | |  |
| 11 | 13 | 21029 | | 5.4 | | 0 | | 0 | | 0.87 | |  |
| 11 | 9 | 6132 | | 1.6 | | 7 | | 4.55 | | 0.89 | |  |
| 11 | 22 | 21569 | | 5.3 | | 0 | | 0 | | 0.9 | |  |
| 11 | 37 | 19595 | | 5.3 | | 0 | | 0 | | 0.91 | |  |
| 11 | 3 | 850 | | 0.2 | | 3 | | 13.53 | | 0.91 | |  |
| 11 | 3 | 8887 | | 2.2 | | 8 | | 3.58 | | 0.99 | |  |
| 11 | 8 | 19979 | | 4.8 | | 0 | | 0 | | 0.99 | |  |
| 11 | 1 | 324 | | 0.1 | | 2 | | 21.5 | | 1 | |  |
| 11 | 8 | 17225 | | 4.4 | | 0 | | 0 | | 1 | |  |
| 11 | 1 | 4570 | | 1 | | 5 | | 4.91 | | 1 | |  |
| 11 | 2 | 1356 | | 0.4 | | 3 | | 8.2 | | 1 | |  |
| 12 | 39 | 425342 | | 103.9 | | 147 | | 1.48 | | 0.07 | |  |
| 12 | 94 | 234976 | | 59.3 | | 33 | | 0.54 | | 0.36 | |  |
| 12 | 26 | 27364 | | 6.6 | | 0 | | 0 | | 0.54 | |  |
| 12 | 9 | 6062 | | 1.5 | | 7 | | 4.64 | | 0.86 | |  |
| 12 | 3 | 833 | | 0.2 | | 3 | | 13.99 | | 0.89 | |  |
| 12 | 13 | 21020 | | 5.4 | | 0 | | 0 | | 0.92 | |  |
| 12 | 22 | 21538 | | 5.3 | | 0 | | 0 | | 0.93 | |  |
| 12 | 37 | 19494 | | 5.2 | | 0 | | 0 | | 0.94 | |  |
| 12 | 10 | 20138 | | 4.9 | | 0 | | 0 | | 0.96 | |  |
| 12 | 3 | 8932 | | 2.2 | | 8 | | 3.58 | | 0.98 | |  |
| 12 | 1 | 320 | | 0.1 | | 2 | | 22.09 | | 0.99 | |  |
| 12 | 8 | 17251 | | 4.4 | | 0 | | 0 | | 0.99 | |  |
| 12 | 1 | 4545 | | 1 | | 5 | | 4.91 | | 1 | |  |
| 13 | 66 | 330928 | | 81.9 | | 118 | | 1.5 | | 0.25 | |  |
| 13 | 94 | 235936 | | 59.1 | | 33 | | 0.54 | | 0.43 | |  |
| 13 | 26 | 27314 | | 6.6 | | 0 | | 0 | | 0.58 | |  |
| 13 | 3 | 812 | | 0.2 | | 3 | | 14.46 | | 0.89 | |  |
| 13 | 13 | 21008 | | 5.3 | | 0 | | 0 | | 0.91 | |  |
| 13 | 22 | 21506 | | 5.3 | | 0 | | 0 | | 0.94 | |  |
| 13 | 37 | 19410 | | 5.1 | | 0 | | 0 | | 0.97 | |  |
| 13 | 10 | 20011 | | 4.8 | | 0 | | 0 | | 0.99 | |  |
| 14 | 62 | 356773 | | 87.7 | | 125 | | 1.48 | | 0.22 | |  |
| 14 | 94 | 236927 | | 58.9 | | 33 | | 0.55 | | 0.38 | |  |
| 14 | 26 | 27270 | | 6.5 | | 0 | | 0 | | 0.6 | |  |
| 14 | 3 | 793 | | 0.2 | | 3 | | 15.04 | | 0.84 | |  |
| 14 | 13 | 20987 | | 5.3 | | 0 | | 0 | | 0.92 | |  |
| 14 | 22 | 21490 | | 5.2 | | 0 | | 0 | | 0.92 | |  |
| 14 | 37 | 19325 | | 5 | | 0 | | 0 | | 0.97 | |  |
| 14 | 3 | 9015 | | 2.2 | | 8 | | 3.59 | | 0.98 | |  |
| 14 | 1 | 313 | | 0.1 | | 2 | | 23.18 | | 0.98 | |  |
| 14 | 10 | 19898 | | 4.8 | | 0 | | 0 | | 0.99 | |  |
| 15 | 57 | 299359 | | 73.9 | | 109 | | 1.53 | | 0.17 | |  |
| 15 | 152 | 313274 | | 76.8 | | 47 | | 0.59 | | 0.35 | |  |
| 15 | 26 | 27218 | | 6.5 | | 0 | | 0 | | 0.56 | |  |
| 15 | 3 | 776 | | 0.2 | | 3 | | 15.66 | | 0.83 | |  |
| 15 | 13 | 20968 | | 5.2 | | 0 | | 0 | | 0.9 | |  |
| 15 | 22 | 21483 | | 5.2 | | 0 | | 0 | | 0.9 | |  |
| 15 | 37 | 19257 | | 4.8 | | 0 | | 0 | | 0.96 | |  |
| 15 | 10 | 19791 | | 4.7 | | 0 | | 0 | | 0.96 | |  |
| 15 | 3 | 9053 | | 2.2 | | 8 | | 3.6 | | 0.97 | |  |
| 15 | 1 | 312 | | 0.1 | | 2 | | 23.97 | | 0.98 | |  |
| 15 | 13 | 31020 | | 7.3 | | 1 | | 0.14 | | 0.99 | |  |
| 15 | 8 | 17367 | | 4.3 | | 0 | | 0 | | 0.99 | |  |
| 15 | 16 | 19890 | | 4.6 | | 12 | | 2.61 | | 1 | |  |
| 16 | 57 | 297521 | | 73.5 | | 109 | | 1.54 | | 0.18 | |  |
| 16 | 94 | 239043 | | 58.2 | | 33 | | 0.55 | | 0.37 | |  |
| 16 | 26 | 27160 | | 6.4 | | 0 | | 0 | | 0.54 | |  |
| 16 | 3 | 758 | | 0.2 | | 3 | | 16.26 | | 0.79 | |  |
| 16 | 2 | 4634 | | 1.1 | | 6 | | 5.26 | | 0.88 | |  |
| 16 | 22 | 21467 | | 5.2 | | 0 | | 0 | | 0.9 | |  |
| 16 | 13 | 20946 | | 5.1 | | 0 | | 0 | | 0.9 | |  |
| 16 | 3 | 988 | | 0.2 | | 3 | | 12.15 | | 0.93 | |  |
| 16 | 1 | 309 | | 0.1 | | 2 | | 24.69 | | 0.96 | |  |
| 16 | 3 | 9089 | | 2.2 | | 8 | | 3.6 | | 0.97 | |  |
| 16 | 16 | 19660 | | 4.6 | | 12 | | 2.65 | | 0.98 | |  |
| 16 | 37 | 19191 | | 4.7 | | 0 | | 0 | | 0.98 | |  |
| 16 | 10 | 19705 | | 4.7 | | 0 | | 0 | | 0.98 | |  |
| 16 | 1 | 1480 | | 0.3 | | 3 | | 8.48 | | 1 | |  |
| 17 | 57 | 295611 | | 72.7 | | 110 | | 1.57 | | 0.09 | |  |
| 17 | 94 | 240224 | | 57.9 | | 33 | | 0.56 | | 0.47 | |  |
| 17 | 16 | 26217 | | 6.4 | | 0 | | 0 | | 0.56 | |  |
| 17 | 26 | 27103 | | 6.4 | | 0 | | 0 | | 0.56 | |  |
| 17 | 3 | 740 | | 0.2 | | 3 | | 16.9 | | 0.79 | |  |
| 17 | 13 | 20910 | | 5.1 | | 0 | | 0 | | 0.9 | |  |
| 17 | 7 | 19841 | | 4.8 | | 0 | | 0 | | 0.94 | |  |
| 17 | 3 | 986 | | 0.2 | | 3 | | 12.29 | | 0.96 | |  |
| 17 | 10 | 19642 | | 4.6 | | 0 | | 0 | | 0.97 | |  |
| 17 | 37 | 19141 | | 4.6 | | 0 | | 0 | | 0.97 | |  |
| 17 | 1 | 307 | | 0.1 | | 2 | | 25.28 | | 0.98 | |  |
| 17 | 3 | 9118 | | 2.2 | | 8 | | 3.62 | | 0.99 | |  |
| 17 | 16 | 19457 | | 4.5 | | 12 | | 2.71 | | 0.99 | |  |
| 18 | 57 | 293853 | | 72.4 | | 108 | | 1.55 | | 0.15 | |  |
| 18 | 94 | 241506 | | 57.8 | | 32 | | 0.54 | | 0.28 | |  |
| 18 | 26 | 27034 | | 6.3 | | 0 | | 0 | | 0.51 | |  |
| 18 | 16 | 26256 | | 6.3 | | 0 | | 0 | | 0.51 | |  |
| 18 | 3 | 730 | | 0.2 | | 3 | | 17.42 | | 0.75 | |  |
| 18 | 13 | 20888 | | 5 | | 0 | | 0 | | 0.95 | |  |
| 18 | 7 | 19849 | | 4.8 | | 0 | | 0 | | 0.97 | |  |
| 18 | 3 | 989 | | 0.2 | | 3 | | 12.4 | | 0.97 | |  |
| 18 | 1 | 308 | | 0.1 | | 2 | | 25.89 | | 0.98 | |  |
| 18 | 3 | 9144 | | 2.2 | | 8 | | 3.61 | | 0.98 | |  |
| 18 | 10 | 19585 | | 4.6 | | 0 | | 0 | | 0.99 | |  |
| 18 | 37 | 19138 | | 4.5 | | 0 | | 0 | | 0.99 | |  |
| 18 | 1 | 4459 | | 1 | | 5 | | 5.01 | | 0.99 | |  |
| 18 | 13 | 30835 | | 7.2 | | 1 | | 0.14 | | 0.99 | |  |
| 18 | 1 | 1462 | | 0.3 | | 3 | | 8.6 | | 1 | |  |
| 19 | 57 | 297116 | | 72.4 | | 108 | | 1.55 | | 0.12 | |  |
| 19 | 94 | 239519 | | 58.4 | | 32 | | 0.53 | | 0.34 | |  |
| 19 | 26 | 27071 | | 6.6 | | 0 | | 0 | | 0.56 | |  |
| 19 | 16 | 26113 | | 6.4 | | 0 | | 0 | | 0.6 | |  |
| 19 | 3 | 726 | | 0.2 | | 3 | | 17.01 | | 0.77 | |  |
| 19 | 1 | 298 | | 0.1 | | 2 | | 27.61 | | 0.96 | |  |
| 19 | 13 | 20885 | | 5.1 | | 0 | | 0 | | 0.97 | |  |
| 19 | 38 | 20866 | | 5.1 | | 0 | | 0 | | 0.97 | |  |
| 19 | 3 | 9162 | | 2.2 | | 8 | | 3.6 | | 0.98 | |  |
| 19 | 7 | 19784 | | 4.8 | | 0 | | 0 | | 0.99 | |  |
| 19 | 8 | 19619 | | 4.8 | | 0 | | 0 | | 0.99 | |  |
| 20 | 94 | 240837 | | 58.6 | | 32 | | 0.53 | | 0.26 | |  |
| 20 | 66 | 322936 | | 78.6 | | 113 | | 1.49 | | 0.28 | |  |
| 20 | 26 | 26853 | | 6.5 | | 0 | | 0 | | 0.52 | |  |
| 20 | 16 | 26110 | | 6.3 | | 0 | | 0 | | 0.59 | |  |
| 20 | 3 | 696 | | 0.2 | | 3 | | 17.77 | | 0.72 | |  |
| 20 | 13 | 20815 | | 5.1 | | 0 | | 0 | | 0.94 | |  |
| 20 | 38 | 20663 | | 5 | | 0 | | 0 | | 0.94 | |  |
| 20 | 7 | 19802 | | 4.8 | | 0 | | 0 | | 0.97 | |  |
| 20 | 8 | 19583 | | 4.8 | | 0 | | 0 | | 0.97 | |  |
| 20 | 3 | 9174 | | 2.2 | | 8 | | 3.6 | | 0.98 | |  |
| 20 | 13 | 30880 | | 7.5 | | 1 | | 0.13 | | 1 | |  |
| 21 | 94 | 242199 | | 58.4 | | 32 | | 0.53 | | 0.25 | |  |
| 21 | 12 | 68900 | | 16.6 | | 34 | | 2.08 | | 0.28 | |  |
| 21 | 26 | 26654 | | 6.4 | | 0 | | 0 | | 0.58 | |  |
| 21 | 16 | 26117 | | 6.3 | | 0 | | 0 | | 0.62 | |  |
| 21 | 3 | 671 | | 0.2 | | 3 | | 18.59 | | 0.67 | |  |
| 21 | 13 | 20757 | | 5 | | 0 | | 0 | | 0.93 | |  |
| 21 | 38 | 20513 | | 5 | | 0 | | 0 | | 0.94 | |  |
| 21 | 7 | 19822 | | 4.8 | | 0 | | 0 | | 0.96 | |  |
| 21 | 2 | 58936 | | 14.2 | | 27 | | 1.92 | | 0.97 | |  |
| 21 | 8 | 19558 | | 4.7 | | 0 | | 0 | | 0.98 | |  |
| 21 | 3 | 9180 | | 2.2 | | 8 | | 3.63 | | 0.98 | |  |
| 21 | 13 | 30808 | | 7.4 | | 1 | | 0.13 | | 0.99 | |  |
| 22 | 12 | 68116 | | 16.3 | | 34 | | 2.12 | | 0.2 | |  |
| 22 | 94 | 243514 | | 58.3 | | 32 | | 0.53 | | 0.29 | |  |
| 22 | 26 | 26523 | | 6.3 | | 0 | | 0 | | 0.54 | |  |
| 22 | 16 | 26153 | | 6.3 | | 0 | | 0 | | 0.58 | |  |
| 22 | 3 | 653 | | 0.2 | | 3 | | 19.25 | | 0.62 | |  |
| 22 | 3 | 22879 | | 5.5 | | 0 | | 0 | | 0.85 | |  |
| 22 | 13 | 20718 | | 5 | | 0 | | 0 | | 0.95 | |  |
| 22 | 38 | 20456 | | 4.9 | | 0 | | 0 | | 0.97 | |  |
| 22 | 7 | 19851 | | 4.8 | | 0 | | 0 | | 0.98 | |  |
| 22 | 3 | 9187 | | 2.2 | | 8 | | 3.66 | | 0.98 | |  |
| 22 | 8 | 19545 | | 4.7 | | 0 | | 0 | | 0.99 | |  |
| 22 | 13 | 30730 | | 7.3 | | 1 | | 0.14 | | 0.99 | |  |
| 23 | 94 | 244833 | | 58.1 | | 32 | | 0.54 | | 0.29 | |  |
| 23 | 12 | 67385 | | 16 | | 33 | | 2.1 | | 0.44 | |  |
| 23 | 26 | 26500 | | 6.3 | | 0 | | 0 | | 0.54 | |  |
| 23 | 16 | 26249 | | 6.2 | | 0 | | 0 | | 0.56 | |  |
| 23 | 3 | 650 | | 0.1 | | 3 | | 19.48 | | 0.62 | |  |
| 23 | 3 | 22703 | | 5.4 | | 0 | | 0 | | 0.85 | |  |
| 23 | 2 | 58648 | | 13.9 | | 27 | | 1.96 | | 0.93 | |  |
| 23 | 61 | 92648 | | 22 | | 9 | | 0.4 | | 0.93 | |  |
| 23 | 38 | 20569 | | 4.9 | | 0 | | 0 | | 0.95 | |  |
| 23 | 3 | 9191 | | 2.2 | | 8 | | 3.69 | | 0.96 | |  |
| 23 | 7 | 19883 | | 4.7 | | 0 | | 0 | | 0.97 | |  |
| 23 | 13 | 30654 | | 7.3 | | 1 | | 0.14 | | 0.99 | |  |
| 24 | 62 | 338839 | | 80.1 | | 115 | | 1.49 | | 0.27 | |  |
| 24 | 129 | 279236 | | 66 | | 38 | | 0.56 | | 0.34 | |  |
| 24 | 3 | 660 | | 0.2 | | 3 | | 19.29 | | 0.6 | |  |
| 24 | 26 | 26547 | | 6.3 | | 0 | | 0 | | 0.61 | |  |
| 24 | 16 | 26362 | | 6.2 | | 0 | | 0 | | 0.61 | |  |
| 24 | 3 | 22605 | | 5.3 | | 0 | | 0 | | 0.86 | |  |
| 24 | 61 | 92863 | | 22 | | 9 | | 0.4 | | 0.92 | |  |
| 24 | 7 | 19913 | | 4.7 | | 0 | | 0 | | 0.96 | |  |
| 24 | 35 | 19385 | | 4.6 | | 0 | | 0 | | 0.97 | |  |
| 24 | 3 | 9196 | | 2.2 | | 8 | | 3.7 | | 0.98 | |  |
| 25 | 12 | 66359 | | 15.6 | | 33 | | 2.16 | | 0.19 | |  |
| 25 | 26 | 26625 | | 6.2 | | 0 | | 0 | | 0.6 | |  |
| 25 | 16 | 26458 | | 6.2 | | 0 | | 0 | | 0.6 | |  |
| 25 | 129 | 280337 | | 65.7 | | 40 | | 0.59 | | 0.6 | |  |
| 25 | 3 | 669 | | 0.2 | | 3 | | 19.2 | | 0.62 | |  |
| 25 | 3 | 22546 | | 5.3 | | 0 | | 0 | | 0.84 | |  |
| 25 | 38 | 20996 | | 4.9 | | 0 | | 0 | | 0.96 | |  |
| 25 | 61 | 93089 | | 21.8 | | 9 | | 0.41 | | 0.96 | |  |
| 25 | 3 | 9200 | | 2.2 | | 8 | | 3.73 | | 0.96 | |  |
| 25 | 2 | 58261 | | 13.7 | | 26 | | 1.93 | | 0.98 | |  |
| 25 | 7 | 19939 | | 4.7 | | 0 | | 0 | | 0.98 | |  |
| 25 | 19 | 112937 | | 26.5 | | 42 | | 1.61 | | 1 | |  |
| 25 | 7 | 10379 | | 2.4 | | 8 | | 3.31 | | 1 | |  |
| 25 | 1 | 1441 | | 0.3 | | 3 | | 8.91 | | 1 | |  |
| 25 | 1 | 4456 | | 1 | | 5 | | 4.81 | | 1 | |  |
| 26 | 12 | 65708 | | 15.3 | | 34 | | 2.26 | | 0.12 | |  |
| 26 | 26 | 26863 | | 6.3 | | 0 | | 0 | | 0.63 | |  |
| 26 | 16 | 26670 | | 6.2 | | 0 | | 0 | | 0.65 | |  |
| 26 | 129 | 282022 | | 65.7 | | 40 | | 0.59 | | 0.65 | |  |
| 26 | 3 | 698 | | 0.2 | | 3 | | 18.48 | | 0.68 | |  |
| 26 | 3 | 22538 | | 5.2 | | 0 | | 0 | | 0.9 | |  |
| 26 | 38 | 21469 | | 5 | | 0 | | 0 | | 0.95 | |  |
| 26 | 13 | 20871 | | 4.9 | | 0 | | 0 | | 0.95 | |  |
| 26 | 7 | 19929 | | 4.7 | | 0 | | 0 | | 0.96 | |  |
| 26 | 3 | 9206 | | 2.1 | | 8 | | 3.75 | | 0.96 | |  |
| 27 | 12 | 64975 | | 15.1 | | 34 | | 2.3 | | 0.08 | |  |
| 27 | 26 | 27094 | | 6.3 | | 0 | | 0 | | 0.6 | |  |
| 27 | 16 | 26887 | | 6.2 | | 0 | | 0 | | 0.6 | |  |
| 27 | 129 | 283567 | | 65.9 | | 40 | | 0.59 | | 0.6 | |  |
| 27 | 3 | 731 | | 0.2 | | 3 | | 17.71 | | 0.73 | |  |
| 27 | 3 | 22559 | | 5.2 | | 0 | | 0 | | 0.89 | |  |
| 27 | 38 | 21955 | | 5.1 | | 0 | | 0 | | 0.92 | |  |
| 27 | 3 | 9214 | | 2.1 | | 8 | | 3.76 | | 0.95 | |  |
| 27 | 13 | 20960 | | 4.9 | | 0 | | 0 | | 0.98 | |  |
| 27 | 7 | 19878 | | 4.6 | | 0 | | 0 | | 0.99 | |  |
| 27 | 2 | 57347 | | 13.3 | | 25 | | 1.9 | | 1 | |  |
| 28 | 12 | 64094 | | 14.8 | | 32 | | 2.2 | | 0.24 | |  |
| 28 | 129 | 284984 | | 65.9 | | 39 | | 0.58 | | 0.44 | |  |
| 28 | 26 | 27349 | | 6.3 | | 0 | | 0 | | 0.62 | |  |
| 28 | 16 | 27135 | | 6.3 | | 0 | | 0 | | 0.62 | |  |
| 28 | 36 | 26974 | | 6.2 | | 0 | | 0 | | 0.64 | |  |
| 28 | 3 | 771 | | 0.2 | | 3 | | 16.88 | | 0.74 | |  |
| 28 | 5 | 10088 | | 2.3 | | 9 | | 3.88 | | 0.8 | |  |
| 28 | 3 | 22595 | | 5.2 | | 0 | | 0 | | 0.89 | |  |
| 28 | 13 | 21064 | | 4.9 | | 0 | | 0 | | 0.96 | |  |
| 28 | 7 | 19781 | | 4.6 | | 0 | | 0 | | 0.96 | |  |
| 28 | 13 | 30468 | | 7 | | 1 | | 0.14 | | 0.99 | |  |
| 29 | 12 | 63050 | | 14.5 | | 30 | | 2.11 | | 0.46 | |  |
| 29 | 129 | 286394 | | 65.7 | | 39 | | 0.58 | | 0.46 | |  |
| 29 | 26 | 27608 | | 6.3 | | 0 | | 0 | | 0.5 | |  |
| 29 | 36 | 27541 | | 6.3 | | 0 | | 0 | | 0.51 | |  |
| 29 | 16 | 27408 | | 6.3 | | 0 | | 0 | | 0.53 | |  |
| 29 | 1 | 1510 | | 0.3 | | 4 | | 11.59 | | 0.59 | |  |
| 29 | 3 | 22660 | | 5.2 | | 0 | | 0 | | 0.92 | |  |
| 29 | 13 | 21172 | | 4.8 | | 0 | | 0 | | 0.96 | |  |
| 29 | 3 | 9211 | | 2.1 | | 8 | | 3.81 | | 0.97 | |  |
| 29 | 7 | 19646 | | 4.5 | | 0 | | 0 | | 0.98 | |  |
| 29 | 19 | 109769 | | 25.2 | | 41 | | 1.66 | | 0.99 | |  |
| 29 | 13 | 30446 | | 7 | | 1 | | 0.14 | | 0.99 | |  |
| 30 | 138 | 298709 | | 68.1 | | 41 | | 0.59 | | 0.59 | |  |
| 30 | 36 | 28101 | | 6.4 | | 0 | | 0 | | 0.61 | |  |
| 30 | 26 | 27821 | | 6.3 | | 0 | | 0 | | 0.64 | |  |
| 30 | 44 | 373601 | | 85.2 | | 118 | | 1.44 | | 0.67 | |  |
| 30 | 7 | 27198 | | 6.2 | | 0 | | 0 | | 0.67 | |  |
| 30 | 3 | 9199 | | 2.1 | | 8 | | 3.83 | | 0.92 | |  |
| 30 | 19 | 108785 | | 24.8 | | 41 | | 1.68 | | 0.97 | |  |
| 30 | 13 | 21276 | | 4.8 | | 0 | | 0 | | 0.99 | |  |
| 31 | 55 | 243176 | | 55 | | 83 | | 1.55 | | 0.48 | |  |
| 31 | 129 | 288735 | | 65.3 | | 39 | | 0.58 | | 0.49 | |  |
| 31 | 36 | 28657 | | 6.5 | | 0 | | 0 | | 0.49 | |  |
| 31 | 26 | 28011 | | 6.3 | | 0 | | 0 | | 0.56 | |  |
| 31 | 1 | 1529 | | 0.3 | | 4 | | 11.6 | | 0.59 | |  |
| 31 | 7 | 26953 | | 6.1 | | 0 | | 0 | | 0.61 | |  |
| 31 | 3 | 9184 | | 2.1 | | 8 | | 3.87 | | 0.93 | |  |
| 31 | 19 | 22367 | | 5.1 | | 0 | | 0 | | 0.98 | |  |
| 31 | 19 | 107834 | | 24.4 | | 40 | | 1.67 | | 0.99 | |  |
| 32 | 138 | 300913 | | 67.7 | | 40 | | 0.57 | | 0.4 | |  |
| 32 | 36 | 29104 | | 6.5 | | 0 | | 0 | | 0.6 | |  |
| 32 | 26 | 28129 | | 6.3 | | 0 | | 0 | | 0.68 | |  |
| 32 | 1 | 1534 | | 0.3 | | 4 | | 11.64 | | 0.69 | |  |
| 32 | 55 | 241164 | | 54.2 | | 81 | | 1.54 | | 0.69 | |  |
| 32 | 7 | 26719 | | 6 | | 0 | | 0 | | 0.76 | |  |
| 32 | 3 | 9171 | | 2.1 | | 8 | | 3.9 | | 0.93 | |  |
| 32 | 19 | 22572 | | 5.1 | | 0 | | 0 | | 0.98 | |  |
| 32 | 2 | 2501 | | 0.6 | | 4 | | 7.14 | | 0.99 | |  |
| 32 | 13 | 21479 | | 4.8 | | 0 | | 0 | | 0.99 | |  |
| 33 | 138 | 301681 | | 67.5 | | 40 | | 0.58 | | 0.42 | |  |
| 33 | 36 | 29378 | | 6.6 | | 0 | | 0 | | 0.51 | |  |
| 33 | 26 | 28134 | | 6.3 | | 0 | | 0 | | 0.62 | |  |
| 33 | 55 | 239146 | | 53.5 | | 80 | | 1.54 | | 0.69 | |  |
| 33 | 7 | 26528 | | 5.9 | | 0 | | 0 | | 0.73 | |  |
| 33 | 3 | 9160 | | 2 | | 8 | | 3.93 | | 0.92 | |  |
| 33 | 19 | 22663 | | 5.1 | | 0 | | 0 | | 0.94 | |  |
| 33 | 13 | 21528 | | 4.8 | | 0 | | 0 | | 0.98 | |  |
| 34 | 55 | 236847 | | 52.3 | | 81 | | 1.6 | | 0.41 | |  |
| 34 | 8 | 30401 | | 6.7 | | 0 | | 0 | | 0.44 | |  |
| 34 | 36 | 29592 | | 6.5 | | 0 | | 0 | | 0.51 | |  |
| 34 | 152 | 334548 | | 73.9 | | 46 | | 0.6 | | 0.52 | |  |
| 34 | 26 | 28067 | | 6.2 | | 0 | | 0 | | 0.67 | |  |
| 34 | 10 | 27413 | | 6.1 | | 0 | | 0 | | 0.76 | |  |
| 34 | 12 | 24317 | | 5.4 | | 0 | | 0 | | 0.9 | |  |
| 34 | 3 | 9148 | | 2 | | 8 | | 3.98 | | 0.93 | |  |
| 34 | 19 | 22693 | | 5 | | 0 | | 0 | | 0.97 | |  |
| 34 | 13 | 21556 | | 4.8 | | 0 | | 0 | | 0.98 | |  |
| 34 | 64 | 186522 | | 41.2 | | 61 | | 1.51 | | 0.99 | |  |
| 35 | 65 | 346211 | | 76.1 | | 110 | | 1.5 | | 0.27 | |  |
| 35 | 84 | 225415 | | 49.6 | | 26 | | 0.51 | | 0.38 | |  |
| 35 | 8 | 30341 | | 6.7 | | 0 | | 0 | | 0.5 | |  |
| 35 | 26 | 27989 | | 6.2 | | 0 | | 0 | | 0.69 | |  |
| 35 | 10 | 27302 | | 6 | | 0 | | 0 | | 0.74 | |  |
| 35 | 3 | 9134 | | 2 | | 8 | | 4.01 | | 0.88 | |  |
| 35 | 12 | 24419 | | 5.4 | | 0 | | 0 | | 0.88 | |  |
| 35 | 19 | 22726 | | 5 | | 0 | | 0 | | 0.93 | |  |
| 35 | 13 | 21590 | | 4.8 | | 0 | | 0 | | 0.96 | |  |
| 35 | 64 | 185447 | | 40.8 | | 61 | | 1.53 | | 0.98 | |  |
| 35 | 1 | 1521 | | 0.3 | | 3 | | 8.99 | | 1 | |  |
| 36 | 67 | 315818 | | 68.8 | | 102 | | 1.54 | | 0.24 | |  |
| 36 | 8 | 30217 | | 6.6 | | 0 | | 0 | | 0.54 | |  |
| 36 | 13 | 30130 | | 6.6 | | 0 | | 0 | | 0.54 | |  |
| 36 | 84 | 225726 | | 49.2 | | 27 | | 0.54 | | 0.65 | |  |
| 36 | 26 | 27919 | | 6.1 | | 0 | | 0 | | 0.7 | |  |
| 36 | 10 | 27178 | | 5.9 | | 0 | | 0 | | 0.76 | |  |
| 36 | 12 | 24523 | | 5.3 | | 0 | | 0 | | 0.9 | |  |
| 36 | 3 | 9121 | | 2 | | 8 | | 4.05 | | 0.91 | |  |
| 36 | 13 | 21631 | | 4.7 | | 0 | | 0 | | 0.99 | |  |
| 36 | 64 | 184082 | | 40.1 | | 60 | | 1.53 | | 1 | |  |
| 37 | 32 | 309101 | | 66.5 | | 98 | | 1.53 | | 0.31 | |  |
| 37 | 94 | 255727 | | 55 | | 31 | | 0.55 | | 0.58 | |  |
| 37 | 13 | 30060 | | 6.5 | | 0 | | 0 | | 0.59 | |  |
| 37 | 8 | 30027 | | 6.5 | | 0 | | 0 | | 0.6 | |  |
| 37 | 26 | 27859 | | 6 | | 0 | | 0 | | 0.76 | |  |
| 37 | 10 | 27043 | | 5.8 | | 0 | | 0 | | 0.79 | |  |
| 37 | 12 | 24631 | | 5.3 | | 0 | | 0 | | 0.93 | |  |
| 37 | 17 | 33235 | | 7.2 | | 17 | | 2.4 | | 0.97 | |  |
| 37 | 16 | 22275 | | 4.8 | | 0 | | 0 | | 0.98 | |  |
| 37 | 13 | 21678 | | 4.7 | | 0 | | 0 | | 0.99 | |  |
| 38 | 94 | 255913 | | 54.4 | | 30 | | 0.54 | | 0.41 | |  |
| 38 | 13 | 29987 | | 6.4 | | 0 | | 0 | | 0.6 | |  |
| 38 | 8 | 29762 | | 6.3 | | 0 | | 0 | | 0.63 | |  |
| 38 | 16 | 28994 | | 6.2 | | 0 | | 0 | | 0.67 | |  |
| 38 | 32 | 301170 | | 64.1 | | 92 | | 1.48 | | 0.72 | |  |
| 38 | 16 | 22364 | | 4.8 | | 0 | | 0 | | 0.97 | |  |
| 38 | 21 | 21857 | | 4.7 | | 0 | | 0 | | 0.98 | |  |
| 38 | 13 | 21732 | | 4.6 | | 0 | | 0 | | 0.98 | |  |
| 39 | 94 | 255869 | | 54 | | 30 | | 0.54 | | 0.41 | |  |
| 39 | 2 | 69172 | | 14.6 | | 30 | | 2.09 | | 0.52 | |  |
| 39 | 13 | 29907 | | 6.3 | | 0 | | 0 | | 0.52 | |  |
| 39 | 8 | 29390 | | 6.2 | | 0 | | 0 | | 0.56 | |  |
| 39 | 16 | 29177 | | 6.2 | | 0 | | 0 | | 0.57 | |  |
| 39 | 16 | 22451 | | 4.7 | | 0 | | 0 | | 0.97 | |  |
| 39 | 21 | 21816 | | 4.6 | | 0 | | 0 | | 0.98 | |  |
| 39 | 13 | 21797 | | 4.6 | | 0 | | 0 | | 0.98 | |  |
| 39 | 41 | 35886 | | 7.6 | | 1 | | 0.13 | | 0.99 | |  |
| 40 | 2 | 66736 | | 13.8 | | 31 | | 2.29 | | 0.17 | |  |
| 40 | 13 | 29827 | | 6.2 | | 0 | | 0 | | 0.65 | |  |
| 40 | 16 | 29365 | | 6.1 | | 0 | | 0 | | 0.68 | |  |
| 40 | 8 | 28950 | | 6 | | 0 | | 0 | | 0.75 | |  |
| 40 | 16 | 27862 | | 5.8 | | 0 | | 0 | | 0.81 | |  |
| 40 | 3 | 9069 | | 1.9 | | 8 | | 4.3 | | 0.81 | |  |
| 40 | 82 | 151121 | | 31.2 | | 15 | | 0.47 | | 0.92 | |  |
| 40 | 16 | 22534 | | 4.7 | | 0 | | 0 | | 0.99 | |  |
| 40 | 11 | 26306 | | 5.4 | | 13 | | 2.41 | | 1 | |  |
| 41 | 2 | 63818 | | 13.1 | | 28 | | 2.18 | | 0.51 | |  |
| 41 | 13 | 29739 | | 6.1 | | 0 | | 0 | | 0.67 | |  |
| 41 | 16 | 29573 | | 6.1 | | 0 | | 0 | | 0.68 | |  |
| 41 | 8 | 28396 | | 5.8 | | 0 | | 0 | | 0.73 | |  |
| 41 | 38 | 43880 | | 9 | | 1 | | 0.11 | | 0.73 | |  |
| 41 | 16 | 28120 | | 5.8 | | 0 | | 0 | | 0.75 | |  |
| 42 | 28 | 36380 | | 7.4 | | 0 | | 0 | | 0.3 | |  |
| 42 | 2 | 60573 | | 12.3 | | 27 | | 2.23 | | 0.45 | |  |
| 42 | 16 | 29790 | | 6 | | 0 | | 0 | | 0.68 | |  |
| 42 | 13 | 29648 | | 6 | | 0 | | 0 | | 0.71 | |  |
| 42 | 16 | 28391 | | 5.8 | | 0 | | 0 | | 0.74 | |  |
| 42 | 8 | 27758 | | 5.6 | | 0 | | 0 | | 0.77 | |  |
| 42 | 204 | 568545 | | 115.6 | | 84 | | 0.7 | | 0.85 | |  |
| 42 | 13 | 22042 | | 4.5 | | 0 | | 0 | | 0.99 | |  |
| 42 | 21 | 21746 | | 4.4 | | 0 | | 0 | | 0.99 | |  |
| 43 | 28 | 36256 | | 7.3 | | 0 | | 0 | | 0.34 | |  |
| 43 | 2 | 56930 | | 11.4 | | 26 | | 2.31 | | 0.34 | |  |
| 43 | 16 | 30023 | | 6 | | 0 | | 0 | | 0.67 | |  |
| 43 | 13 | 29552 | | 5.9 | | 0 | | 0 | | 0.68 | |  |
| 43 | 61 | 100776 | | 20.3 | | 7 | | 0.34 | | 0.68 | |  |
| 43 | 8 | 27024 | | 5.4 | | 0 | | 0 | | 0.84 | |  |
| 43 | 37 | 42022 | | 8.4 | | 1 | | 0.12 | | 0.86 | |  |
| 43 | 17 | 31927 | | 6.4 | | 15 | | 2.36 | | 0.99 | |  |
| 43 | 16 | 22779 | | 4.6 | | 0 | | 0 | | 0.99 | |  |
| 43 | 16 | 37304 | | 7.5 | | 1 | | 0.13 | | 0.99 | |  |
| 44 | 28 | 36128 | | 7.1 | | 0 | | 0 | | 0.35 | |  |
| 44 | 56 | 128528 | | 25.3 | | 10 | | 0.39 | | 0.66 | |  |
| 44 | 28 | 57031 | | 11.2 | | 2 | | 0.18 | | 0.79 | |  |
| 44 | 13 | 29456 | | 5.8 | | 0 | | 0 | | 0.79 | |  |
| 44 | 8 | 26276 | | 5.2 | | 0 | | 0 | | 0.9 | |  |
| 44 | 3 | 59823 | | 11.8 | | 24 | | 2.07 | | 0.93 | |  |
| 44 | 16 | 22858 | | 4.5 | | 0 | | 0 | | 0.99 | |  |
| 44 | 13 | 22244 | | 4.4 | | 0 | | 0 | | 0.99 | |  |
| 44 | 16 | 36918 | | 7.3 | | 1 | | 0.14 | | 0.99 | |  |
| 44 | 25 | 21636 | | 4.3 | | 0 | | 0 | | 0.99 | |  |
| 45 | 28 | 36001 | | 7 | | 0 | | 0 | | 0.44 | |  |
| 45 | 61 | 102324 | | 19.8 | | 6 | | 0.3 | | 0.51 | |  |
| 45 | 204 | 564318 | | 108.9 | | 76 | | 0.67 | | 0.64 | |  |
| 45 | 13 | 29364 | | 5.7 | | 0 | | 0 | | 0.79 | |  |
| 45 | 8 | 25517 | | 4.9 | | 0 | | 0 | | 0.92 | |  |
| 45 | 12 | 25005 | | 4.8 | | 13 | | 2.72 | | 1 | |  |
| 46 | 274 | 748939 | | 142.5 | | 104 | | 0.69 | | 0.39 | |  |
| 46 | 61 | 103153 | | 19.6 | | 6 | | 0.3 | | 0.54 | |  |
| 46 | 13 | 29297 | | 5.6 | | 0 | | 0 | | 0.86 | |  |
| 46 | 8 | 24822 | | 4.7 | | 0 | | 0 | | 0.98 | |  |
| 46 | 50 | 131869 | | 25.1 | | 41 | | 1.67 | | 1 | |  |
| 47 | 23 | 31366 | | 5.9 | | 0 | | 0 | | 0.72 | |  |
| 47 | 53 | 69418 | | 13 | | 3 | | 0.23 | | 0.78 | |  |
| 47 | 13 | 29257 | | 5.5 | | 0 | | 0 | | 0.8 | |  |
| 47 | 6 | 52533 | | 9.9 | | 21 | | 2.16 | | 0.91 | |  |
| 47 | 3 | 8973 | | 1.7 | | 7 | | 4.18 | | 0.95 | |  |
| 47 | 16 | 26935 | | 5.1 | | 13 | | 2.6 | | 0.98 | |  |
| 47 | 12 | 36910 | | 6.9 | | 16 | | 2.34 | | 0.98 | |  |
| 47 | 19 | 25264 | | 4.7 | | 0 | | 0 | | 0.99 | |  |
| 48 | 6 | 49582 | | 9.1 | | 22 | | 2.45 | | 0.46 | |  |
| 48 | 23 | 31755 | | 5.9 | | 0 | | 0 | | 0.74 | |  |
| 48 | 16 | 30215 | | 5.6 | | 0 | | 0 | | 0.81 | |  |
| 48 | 13 | 29241 | | 5.4 | | 0 | | 0 | | 0.9 | |  |
| 48 | 19 | 25445 | | 4.7 | | 0 | | 0 | | 0.97 | |  |
| 48 | 16 | 26884 | | 5 | | 13 | | 2.65 | | 0.97 | |  |
| 48 | 8 | 23603 | | 4.3 | | 0 | | 0 | | 0.99 | |  |
| 48 | 16 | 23173 | | 4.3 | | 0 | | 0 | | 0.99 | |  |
| 48 | 1 | 3129 | | 0.6 | | 4 | | 6.96 | | 0.99 | |  |
| 49 | 6 | 46932 | | 8.5 | | 22 | | 2.65 | | 0.22 | |  |
| 49 | 23 | 32159 | | 5.8 | | 0 | | 0 | | 0.78 | |  |
| 49 | 16 | 30547 | | 5.5 | | 0 | | 0 | | 0.81 | |  |
| 49 | 13 | 29259 | | 5.3 | | 0 | | 0 | | 0.91 | |  |
| 49 | 162 | 393781 | | 71.2 | | 98 | | 1.44 | | 0.94 | |  |
| 49 | 16 | 26868 | | 4.9 | | 13 | | 2.71 | | 0.97 | |  |
| 49 | 7 | 43341 | | 7.8 | | 1 | | 0.13 | | 0.97 | |  |
| 49 | 19 | 25631 | | 4.6 | | 0 | | 0 | | 0.97 | |  |
| 50 | 6 | 44458 | | 7.8 | | 22 | | 2.87 | | 0.07 | |  |
| 50 | 5 | 8413 | | 1.5 | | 8 | | 5.46 | | 0.33 | |  |
| 50 | 275 | 737960 | | 129.8 | | 96 | | 0.7 | | 0.83 | |  |
| 50 | 16 | 30909 | | 5.4 | | 0 | | 0 | | 0.93 | |  |
| 50 | 16 | 26888 | | 4.7 | | 13 | | 2.78 | | 0.94 | |  |
| 50 | 128 | 308507 | | 54.3 | | 77 | | 1.47 | | 0.97 | |  |
| 50 | 22 | 29603 | | 5.2 | | 0 | | 0 | | 0.98 | |  |
| 50 | 13 | 29314 | | 5.2 | | 0 | | 0 | | 0.98 | |  |
