# Supplementary material for: Presymptomatic geographical distribution of ALS patients suggests the involvement of environmental factors in the disease pathogenesis
Source: J Neurol. 2023 Jul 25;270(11):5475–82. doi: 10.1007/s00415-023-11888-8 (PMC10576667; doi:10.1007/s00415-023-11888-8)
Supplement: Supplementary file 4 — Supplementary file4 (DOC 40 KB) [file 415_2023_11888_MOESM4_ESM.doc]

**Supplementary table 3.** Probabilities of encountering triples of unrelated patients who had lived in the same dwelling only by chance.

| **Resident capacity of dwellings with triple occurrences (percentiles)** | **Resident capacity of dwellings with triple occurrences (inhabitants/dwelling)** | **Residents/dwelling (mean±SD)** | **Percentage of dwellings with respect to the total number in Turin** | **Number of observed triple occurrences** | **Probability of triple occurrences** |
| --- | --- | --- | --- | --- | --- |
| - | - | 4.2±2.1 | 100 | 3 | 0.86 |
| 100 | 254 | 2.6±1.6 | 99.8 | 2 | 0.40 |
| 90 | 102 | 1.4±1.2 | 97.5 | 2 | 0.30 |
| 80 | 70 | 0.8±0.9 | 94.4 | 2 | 0.09 |
| 75 | 64 | 0.6±0.8 | 93.4 | 2 | 0.04 |
| 60 | 45 | 0.4±0.6 | 88.0 | 2 | 0.001 |
| 50 | 33 | - | 81.2 | - | - |

Probabilities have been calculated through a Monte Carlo simulation where patients were randomly allocated an average of 3.6 times in all buildings in Turin (first line). The second line refers to the iterations in which only buildings with comparable resident capacity to those where triple occurrences were observed were selected. In the subsequent lines, the largest buildings were further excluded based on the resident capacity percentiles of buildings with triple occurrences.
